# Research on the influence of team psychological capital on team members’ work performance

**DOI:** 10.3389/fpsyg.2022.1072158

**Published:** 2022-12-13

**Authors:** Yongji Jiao, Xiaoman Zhang, Siwen Lu, Zimo Wu, Yuqian Deng

**Affiliations:** ^1^School of Management, Nanjing University of Posts and Telecommunications, Nanjing, China; ^2^Huishang Bank Maanshan Sub-branch, Maannshan, China

**Keywords:** cross-level analysis, team members’ work performance, team psychological capital, team members’ psychological capital, team support

## Abstract

Team psychological capital is the positive psychological state of a team and has a positive impact on the human resource management and performance management of the team. Team members’ work performance, as a component of team performance, has an important impact on improving team performance. However, there is less cross-level impact analysis between the two. The purpose of this paper is to explore the mechanisms of the cross-level effects of team psychological capital on members’ performance. A paired questionnaire survey was administered to 387 human resource management team members from 86 human resource management teams across China, and SPSS 22.0, AMOS 23.0, and HLM 6.08 software were used to analyze the questionnaire data at the same level and across levels. The test shows that the cross-level positive effect of team psychological capital on team members’ work performance is established, and the cross-level mediating role of team members’ psychological capital and team support between the two is established. It enriches the cross-level research from team to individual, refines and enriches the research on the impact of team psychological capital on individual behavior, and provides new ideas for team management in companies. Therefore, when conducting team management, enterprises can enhance team members’ psychological capital by improving team psychological capital, thereby improving team members’ work performance and team efficiency.

## Introduction

According to a report from the National Bureau of Statistics of China, during the “14th Five-Year Plan” period, with the new round of industrial division of labor and the reorganization of the global trade pattern, China’s economic development shifted from scale growth to improving quality and seizing the new world of the global industrial revolution. It is very important to seize opportunities, promote high-quality development of the industry, and occupy the commanding heights of global industrial development. In this case, the complexity of the business environment makes the competition faced by enterprises more intense, and organizational performance has become a key factor in enhancing a company’s competitive advantage. The improvement of organizational performance not only enables employees to increase wages but also improves organizational efficiency, thereby promoting innovation in production technology. As an integral part of organizational performance, employee performance will also have a significant impact in the process of improving organizational performance. Many studies have shown that team psychological capital plays a vital role in team members’ work performance.

Psychological capital is a positive psychological state and is present when an individual is faced with tasks, performance, and success, which occurs in specific situations and can be enhanced through conscious development, thus increasing individual competitiveness. Luthans classifies psychological capital into four dimensions: self-efficacy, hope, optimism, and resilience. Most scholars believe that team psychological capital is formed and developed based on individual psychological capital; therefore, the dimensions of team psychological capital are consistent with those of individual psychological capital (Luthans et al., 2007). The role of team psychological capital in organizational development is receiving increasing attention, and it has a significant positive impact on human resource development, organizational performance and the ability of teams to innovate. Tang (2020) found a strong strategic and corporate link between PsyCap and HRD, which has important implications for business development (Tang, 2020). A study by Tho and Duc (2021) demonstrated the positive effect of team psychological capital on a team’s ability to innovate (Tho and Duc, 2021).

In the context of the deepening development of the market economy and in the face of fierce market competition, enterprises must improve the cohesion of their teams, enhance the sense of responsibility of team employees and bring into play the convergence effect of team members. As the scale of operations continues to expand and the number of employees continues to increase, enterprises must adjust their team management model according to the stage of development to reduce management costs and improve management efficiency. The objective of this paper is to investigate the cross-level influence of team psychological capital on employee performance and the mediating role of team support and individual psychological capital in between. In turn, this paper provides a reliable way for companies to build psychological capital from a team management perspective and thereby improve the performance of team members.

Ulrich (1996) suggests that the HRM function contributes to strategy implementation and value creation and that HR managers should play four classic roles: administrative expert, change agent, employee advocate and strategic partner. Gao et al. (2016) discuss six roles that HR should play in the Chinese context in the areas of strategy, operations, and people: enabler, supporter, advisor, partner, caretaker, and motivator. As such, human resource managers play an important role in the management and administration of the organization’s business strategy and the achievement of management objectives (Erxiu et al., 2012; Liping and Linying, 2019).

On this basis, scientific and reasonable countermeasures and suggestions are put forward to improve team members’ work performance and enhance the company’s core competitiveness.

## Literature review and research hypothesis

### The influence of team psychological capital on team members’ work performance

Team psychological capital is developed based on individual psychological capital, which can not only show the general individual psychological characteristics of team members but also show the integration and particularity of team psychology. Team psychological capital is a series of psychological qualities and characteristics that can enhance team creativity. It is formed by the collaborative and intensive interaction of team members’ psychological tacit understanding and positive psychological state and then by giving full play to team’s integrated advantages. Related research on psychological capital includes not only the exploration of its antecedent variables but also the exploration of its outcome variables. There has been research on the impact of individual psychological capital on individual performance and the impact of team psychological capital on team performance. In terms of the impact of individual psychological capital on individual performance, Erxiu et al. (2012) used innovation performance as a result variable to explore the impact mechanism of knowledge-based employees’ psychological capital on innovation performance; Yanyun et al. (2019) found that employees’ psychological capital in state-owned construction firms positively influenced their job performance; Bogler and Somech (2019) examined the positive correlation between team psychological capital and organizational citizenship behavior, which is an important part of work performance; and Jinfeng et al. (2021) found that team psychological capital has a positive impact on individual innovative work behavior, which can promote job performance (Rebelo et al., 2018; Bogler and Somech, 2019; Jinfeng et al., 2021; Paliga et al., 2022). In terms of the impact of team psychological capital on team performance, Liping and Linying (2019) found that team psychological capital has a significant impact on team innovation performance. Rebelo et al. (2018) found that team psychological capital positively affects team performance. Paliga et al. (2022) proved the correlative relationship between psychological capital and job performance at both the individual and team levels (Vanno et al., 2014; Xiaoyun, 2019; Yan et al., 2022). At the same time, Xiaoman et al. (2022) show that team-level variables, such as team psychological capital, have a corresponding effect on individual employee-level variables (Okros and Vîrgă, 2022). Team psychological capital has an impact on team members’ work performance that cannot be underestimated.

Accordingly, this article proposes the following hypotheses:

*H1*: Team psychological capital has a significant impact on team members’ work performance.

### The mediating role of team members’ psychological capital

Team psychological capital is developed based on individual psychological capital. At present, most scholars agree that the dimensions of team psychological capital are the same as those of individual psychological capital. Team psychological capital consists of two levels: the individual level and the group level. The four dimensions of team psychological capital correspond to and are positively correlated with the four dimensions of individual psychological capital (Vanno et al., 2014).

Accordingly, this article proposes the following hypotheses:

*H2*: Team psychological capital is significantly positively correlated with team members’ psychological capital.

There is a significant positive correlation between the psychological capital and work performance of managers of state-owned enterprises (Xiaoyun, 2019). Airline companies should focus more attention on team members’ psychological capital, which is beneficial for their performance (Yan et al., 2022). The same conclusion was reached not only by Romanian correctional officers (Okros and Vîrgă, 2022) but also by employees from seven five-star hotels in China (Huang et al., 2021). Among them, the self-efficacy and resilience dimensions of psychological capital showed a strong positive correlation with the related dimensions of work performance. Relationship. The formation of intrinsic motivation of knowledge workers is positively affected by psychological capital, and it plays a mediating role in the relationship between knowledge workers’ psychological capital and innovation performance; learning, relational, innovative psychological capital, and task-based psychological capital are positively correlated. In research on the influence of subordinates’ psychological capital on the relationships between transformational leadership and satisfaction, subordinates’ psychological capital was found to play a mediating role (Yang et al., 2012). The higher the subordinate’s perception of procedural fairness is, the more likely that his or her psychological capital will be positively affected by transformational leadership. The greater the value is, the stronger the transmission effect of psychological capital between transformational leadership behavior and the performance and satisfaction of subordinates.

Accordingly, this article proposes the following hypotheses:

*H3*: Team members’ psychological capital is significantly positively correlated with team members’ work performance.

*H4*: Team members’ psychological capital plays a mediating role in team psychological capital and team members’ work performance.

### The mediating role of team support

Organizational support evolves into team support, and the essence of team support is organizational support. Organizational support has a positive impact on psychological capital, which plays a mediating role between organizational support and work engagement (Jinhong and Hai, 2017). When observing the new generation of skilled workers in emerging industries, scholars have found that when they feel stronger organizational support, the positive correlation between psychological capital and work-life balance is stronger (Zhongze et al., 2019). Marashdah and Albdareen (2020) found that organizational support has a significant impact on team members’ psychological capital (Marashdah and Albdareen, 2020). Bilgetürk and Baykal (2021) proposed that perceived organizational support will have a positive effect on the psychological capital of individuals (Bilgetürk and Baykal, 2021). Yuan et al. (2022) found that psychological capital has a positive correlation with perceptions of organizational support (Yuan et al., 2022).

Accordingly, this article proposes the following hypotheses:

*H5*: Team psychological capital is significantly positively correlated with team support.

Several studies support the direct correlation between team support and team employees’ job performance from different perspectives. Research by Fu (2019) shows that organizational support has a significant impact on employee performance (Fu, 2019). Xiaohui and Qun (2020) found that organizational support is significantly positively correlated with employee innovation performance (Xiaohui and Qun, 2020). Coll and Mignonac (2022) proposed that perceptions of organizational support by the disability group will yield higher levels of task performance (Coll and Mignonac, 2022).

Niantao and Xiangmin (2019) pointed out that the organizational support plays a moderating role in the relationship between the psychological capital and job satisfaction of hotel management interns (Niantao and Xiangmin, 2019). Li’s (2022) research on scientific researchers found that their psychological capital and innovation performance were regulated by organizational support (Li, 2022).

Accordingly, this article proposes the following hypotheses:

*H6*: Team support is significantly positively correlated with team members’ work performance.

*H7*: Team support plays a mediating role in team psychological capital and team members’ work performance.

## Methodology

### Data collection and reliability testing

This research adopts the paired questionnaire method, which administers an individual questionnaire and a team questionnaire. First, the team-level questionnaires were numbered separately from the individual-level questionnaires. The numbering logic was that the team questionnaires were numbered 001, 002, and 003, while the first individual questionnaire within the team was numbered 001-1, 001-2, and 001-3 and so on. Once the questionnaires are numbered, the two questionnaires are sent to the leader of the HRM team, who organizes the completion of the questionnaires by the members within the team, with the individual questionnaires being completed by the members individually, and the team questionnaires being completed by the leader through discussion with the team members. Therefore, each team has its corresponding number, and this paper can match the team-level questionnaire with the team member-level questionnaire one by one according to this number. Once the questionnaires were completed, the questionnaire software was used to collect the questionnaire data, export the relevant data, and match the questionnaires according to the preprogrammed numbers.

The individual questionnaire includes the measurement of individual psychological capital and individual job performance, which is completed by employees themselves. The team questionnaire includes the measurement of team psychological capital and team support, which is completed by the team through discussion.

This study used the 24-item four-dimensional scale developed by Luthans et al. (2005) to measure team psychological capital and individual psychological capital (Luthans et al., 2005). Regarding the scale of team support, this study used the 17-item three-dimensional scale. Team members’ job performance comprises role performance and organizational citizenship behavior. For the measurement of role performance, this study uses seven scales developed by Williams and Anderson (1991). To measure OCB, this study uses the short scale developed by Farh et al. (2007) in the context of mainland China, which contains three dimensions and nine items in total (Farh et al., 2007).

After testing, the above scales showed high reliability and validity. The Cronbach’s alpha coefficients of team psychological capital, team members’ psychological capital, team members’ work performance, and team support are all >0.8. This indicates that the scale selected in the presurvey is highly reliable and can be used for formal sample surveys. When the reliability of the scale reaches the standard, the validity of the scale needs to be tested by subsequent analysis.

This study not only carries out exploratory factor analysis on the scale but also carries out confirmatory factor analysis. In this study, SPSS 22.0 was used for exploratory factor analysis of the formal questionnaire, and AMOS 23.0 was used for confirmatory factor analysis.

The KMO values of the four scales of team psychological capital, team members’ psychological capital, team support, and team members’ work performance are 0.923, 0.942, 0.913, and 0.894, respectively, which are all >0.8 and are very close to 1, indicating that the above four scales are suitable for factor analysis.

The confirmatory factor analysis results of the second-order four-factor model (the second-order factor is team psychological capital, and the first-order factors are team efficacy, team optimism, team hope, and team toughness) are as follows: *χ*^2^/df = 4.583, NFI = 0.917, RFI = 0.902, CFI = 0.953, and RMSEA = 0.803. The confirmatory factor analysis results of the second-order four-factor model (the second-order factor is team members’ psychological capital, and the first-order factors are self-efficacy, optimism, hope, and resilience) are as follows: *χ*^2^/df = 4.694, NFI = 0.936, RFI = 0.924, CFI = 0.976, and RMSEA = 0.818. The confirmatory factor analysis results of the second-order three-factor model (the second-order factor is team support, and the first-order factors are employee value recognition, organizational support, and concern for benefits) are as follows: *χ*^2^/df = 4.953, GFI = 0.933, RFI = 0.947, CFI = 0.958, and RMSEA = 0.813. The confirmatory factor analysis results of the second-order two-factor model (the second-order factors are team members’ work performance, and the first-order factors are organizational citizenship behavior and in-role performance) are as follows: *χ*^2^/df = 5.259, NFI = 0.913, RFI = 0.921, CFI = 0.956, and RMSEA = 0.809. All four scales show a high degree of construct validity.

### Same level research variable test

For variables at the same level, such as team psychological capital and team support at the team level, the impact of individual team members’ psychological capital and team members’ work performance, AMOS 23.0 software was used for direct analysis. C.R. represents the critical ratio value, which is equivalent to the *t*-value. If the *t*-value is >1.95, the *p*-value is <0.05; if the *t*-value is >2.58, the *p*-value is <0.01. Table 1 shows that the standardized estimated value of team psychological capital for team support is 0.422, and the critical ratio value is 4.718, which is >2.58, indicating that the *p*-value is <0.01. After AMOS 23.0 analysis, the *p*-values of team psychological capital and team support were obtained. If it is <0.001, it satisfies the *p*-value condition required by the critical ratio value. In summary, team psychological capital has a significant impact on team support, and Hypothesis 5 is proven. The standardized estimated value of team members’ psychological capital and team members’ work performance is 0.506, and the critical ratio value is 10.735 greater than 2.58, indicating that the *p*-value is <0.01, greater than the *p*-value in the AMOS 23.0 analysis result, and the *p*-value required to meet the critical ratio value Therefore, team members’ psychological capital has a significant impact on team members’ work performance. Hypothesis 3 is proven.

**Table 1:** Model fitting index table.

	*χ*^2^/df	NFI	RFI	CFI	RMSEA
Team Psychological Capital	4.583	0.917	0.902	0.953	0.803
Team Members’ Psychological Capital	4.694	0.936	0.924	0.976	0.818
Team Support	4.694	0.936	0.924	0.976	0.818
Team Members’ Work Performance	5.259	0.913	0.921	0.956	0.809

### The mediating role of team members’ psychological capital

To verify the two intermediary variables assumed in this study, first, based on the direct effect of team psychological capital on team members’ work performance, team members’ psychological capital and team psychological capital are included in the model at the same time, and the mediating role of team members’ psychological capital is examined. Construct a cross-level indirect interaction model.

The intermediary model of team members’ psychological capital is as follows:

Model 1: Verification of the relationship between team psychological capital and team members’ work performance
Level-1 Model: Team members’ work performance = B0 + R
Level-2 Model: B0 = γ00 + γ01* (team psychological capital) + U0
Model 2: Verification of the relationship between team psychological capital and team members’ psychological capital
Level-1 Model: Team members’ psychological capital = B0 + R
Level-2 Model: B0 = γ00 + γ01* (team psychological capital) + U0
Model 3: Verifying the mediating effect of team members’ psychological capital between team psychological capital and team members’ work performance
Level-1 Model: Employee work performance = B0 + B1* (team members’ psychological capital) + R
Level-2 Model: B0 = γ00 + γ01* (team psychological capital) + γ02* (team members’ psychological capital) + U0
B1 = γ10

The running results of each model are shown in Table 2.

**Table 2:** Model running results parameters.

Level			Parameter estimation			
Path			Standardized estimate	SE	C.R.	Value of *p*
Same level	Team psychological capital → Team support		0.422	0.120	4.718	***
	Team members’ psychological capital → Team members’ work performance		0.506	0.063	10.735	***
Cross-Level	Dependent variable	Model	γ_00_	γ_01_	σ^2^	τ_00_
	Team members’ performance	Zero model	5.226^***^		0.591	0.203^***^
		Team psychological capital → Team members’ work performance	4.138^***^	0.413^***^	0.657	0.076^***^
		Team support →Team members’ work performance	4.578^***^	0.372^***^	0.739	0.041^***^
	Team members’ psychological capital	Zero model	5.433^***^		0.523	0.292^***^
		Team psychological capital → Team members’ psychological capital	4.006^***^	0.363^***^	0.796	0.068^***^

**Figure 1:**
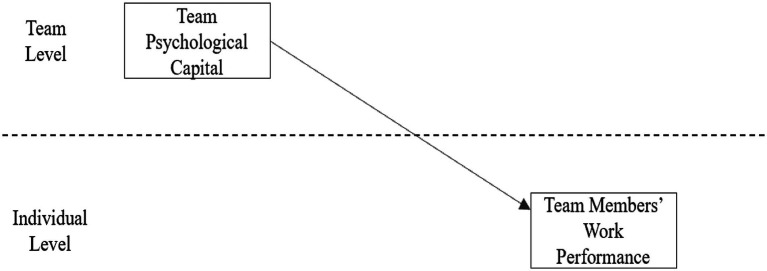
The main effect of team psychological capital and team members’ work performance.

In the HLM 6.08 software, first, the overall level of interpretation of the variable team’s psychological capital to verify the overall effect of the variable team members’ work performance is carried out; that is, the equation of model one is executed, and the result of model one is obtained through the calculation of the HLM 6.08 software. The result is γ01c = 0.413, *p* < 0.001 (shown in Table 1), which shows that team psychological capital has a significant impact on team members’ work performance. Hypothesis 1 is proven. The model is shown in Figure 1.

The intermediary model of team members’ psychological capital as an intermediary variable belongs to 2-1-1. Based on the main effect, the influence of the overall-level variable team psychological capital on the individual-level intermediary variable team members’ psychological capital is verified. Equation, calculated by HLM 6.08 software, provides the result of model two as γ01 = 0.363, *p* < 0.001 (shown in Table 1), which shows that team members’ psychological capital is affected by team psychological capital, and the direction of influence is the same direction. Hypothesis 2 is proven. Finally, we consider both the overall-level explanation variable team psychological capital and the individual-level intermediary variable team members’ psychological capital to test whether the direct effect of team psychological capital on team members’ work performance disappears because of the existence of the intermediary variable team members’ psychological capital, which in turn leads to the production of a complete intermediary effect. We incorporate team psychological capital and team members’ psychological capital into model three at the same time, execute the equation of model three, and calculate with HLM 6.08 software to obtain γ01c,=0.32, *p* > 0.05; γ10b = 0.506, *p* < 0.001, where γ01c represents the effect of team psychological capital and team member’s work performance, and γ10b represents the effect of team member’s psychological capital and team member’s work performance.

**Figure 2:**
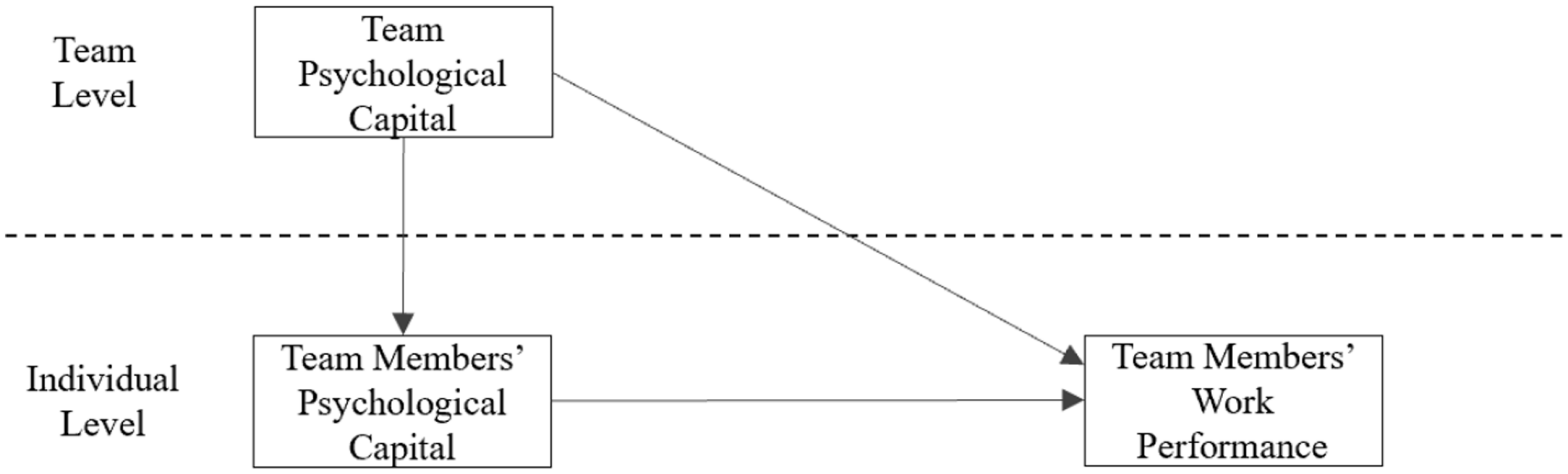
The mediating role of team members’ psychological capital between team psychological capital and team members’ work performance.

According to the model verification results, when the variables team psychological capital and team members’ psychological capital are included in the model at the same time, team members’ psychological capital and team members’ work performance are significantly positively correlated, and the effect of team psychological capital and team members’ work performance is significantly positive. The correlation (γ01c = 0.413, *p* < 0.001) became insignificant (γ01c, =0.32, *p* > 0.05). In summary, team members’ psychological capital plays a completely intermediary role in team psychological capital and team members’ work performance. Hypothesis 3 is proven. Model three is shown in Figure 2.

### The mediating role of team support

For the second mediating variable, team support, based on the main effect, team support and team psychological capital are then included in the model to examine the mediating role of team support. According to the data from Krull and MacKinnon (2001) on the effect of multilevel mediation, the mediation variable team support selected for this study is a 2-2-1 model. The mediation model of team support is as follows:

Model 1: Verification of the relationship between team psychological capital and team members’ work performance
Level-1 Model: Team members’ work performance = B0 + R
Level-2 Model: B0 = γ00 + γ01* (team psychological capital) + U0
Model 4: Verification of the relationship between team psychological capital and team support
Level-2 Model: team support = γ00+ γ01* (team psychological capital) + U0
Model 5: Verification of the mediating effect of team support between team psychological capital and team performance
Level-1 Model: Team members’ work performance = B0+ R
Level-2 Model: B0 = γ00 + γ01* (team psychological capital) + γ02* (team support) + U0

**Figure 3:**
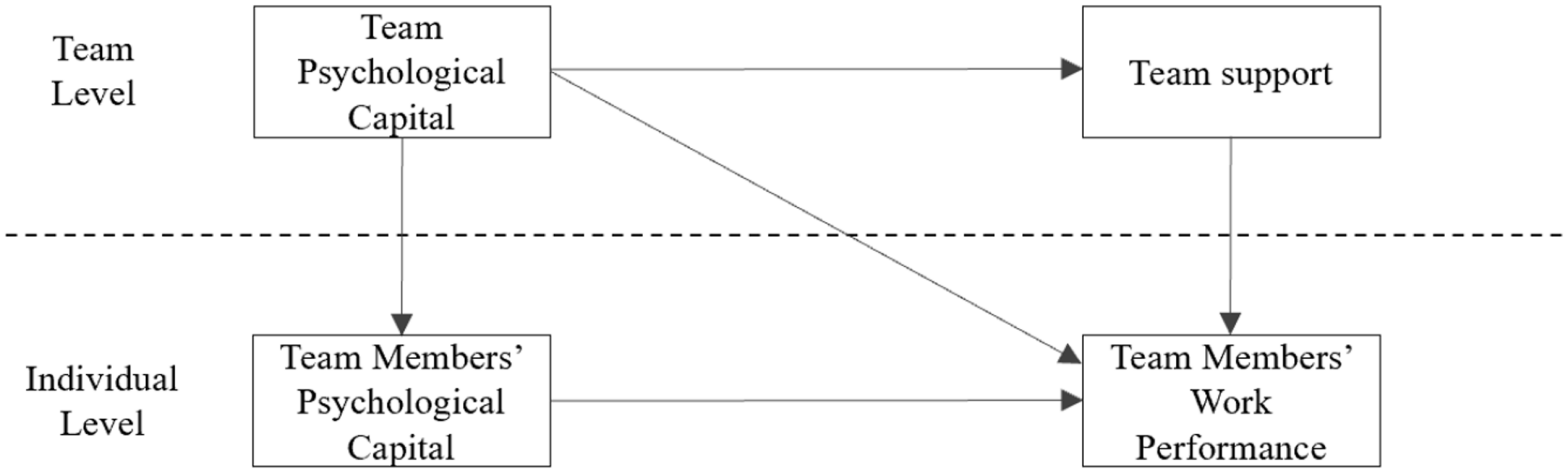
The mediating role of team support between team psychological capital and team members’ work performance.

The mediation model of team support as a mediating variable belongs to 2-2-1. Based on the main effect, it then examines the influence of the overall level variable team psychological capital on the team level mediation variable team support feeling and implements the equation of model 4. After calculation by HLM 6.08 software, the result of model four is obtained. The result of model four is γ01 = 0.422, *p* < 0.001 (shown in Table 1), which shows that team support changes with the change in team psychological capital. The direction is the same, and Hypothesis 5 is further proven. Finally, to consider both the overall level of explanation variable team psychological capital and team-level intermediary variable team support, we also examine whether the direct effect of team psychological capital on team members’ work performance disappears because of the existence of team support, which in turn leads to the production of a complete mediation effect. We incorporate team psychological capital and team support into model five at the same time, execute the equation of the model, and calculate by HLM 6.08 software, obtaining γ01c = 0.18, *p* > 0.05 and γ10b = 0.372, *p* < 0.001, where γ01c represents the effect of team psychological capital and team members’ work performance; γ10b represents the effect of team support and team members’ work performance. According to the model verification results, when the variables of team psychological capital and team support are included in the model, team members’ psychological capital is significantly positively correlated with team members’ work performance. Hypothesis 3 is proven, and team psychological capital is related to team members’ work performance. The effect changed from a significant positive correlation (γ01c = 0.413, *p* < 0.001) to a nonsignificant one (γ01c = 0.18, *p* > 0.05). In summary, team support plays a completely intermediary role in the team’s psychological capital and team members’ work performance, and Hypothesis 7 is proven. Model five is shown in Figure 3.

## Results and discussion

### Positive effect of team psychological capital on team members’ work performance

According to Table 1, team psychological capital has a significant positive impact on team members’ work performance. Therefore, to improve individual work performance, we need to improve team psychological capital. The team’s psychological capital can be effectively improved by establishing staff’s physical and mental health files, setting up psychological education courses, setting up mental health specialists, etc. Team leaders themselves need to consciously and constantly implant, develop and train their own psychological capital; continuously improve their self-management ability; and then pass it on to their subordinates to set a benchmark.

### The mediating role of team members’ psychological capital

According to the model verification results, when the variables team psychological capital and team members’ psychological capital are included in the model at the same time, team members’ psychological capital and team members’ work performance are significantly positively correlated, and the effect of team psychological capital and team members’ work performance is significantly positive. In summary, team members’ psychological capital plays a completely intermediary role in team psychological capital and team members’ work performance, as shown in Figure 2.

To improve team members’ psychological capital, enterprises need to take different measures based on the members’ different educational backgrounds and specifically improve team members’ psychological capital. In addition to the external conditions given by enterprises to enhance psychological capital, the root of promoting individual psychological capital lies in the members themselves. Members need to enhance their sense of self-efficacy, actively learn and update their knowledge structure and skills, take valuable actions toward their goals, and build their interest in their posts and careers.

### The mediating role of team support

According to this article, based on the main effect, team support and team psychological capital are included in the model at the same time, and the mediating effect of team support on team psychological capital and team members’ work performance is established.

To enhance team support of the human resource management team, starting from the three dimensions of team support is necessary, namely, organizational support, value recognition and interest concern. The enterprise needs to clarify the system and specification, give attention to the working environment of human resources personnel, improve the distribution of benefits such as salary and welfare, improve its performance appraisal indicators, link the personal interests of team members with the interests of the team in the performance appraisal, encourage employees through incentives and other means, enhance team support, and then improve team members’ work performance.

## Data availability statement

The original contributions presented in the study are included in the article/supplementary material, further inquiries can be directed to the corresponding author.

## Ethics statement

Written informed consent was obtained from the individual(s) for the publication of any potentially identifiable images or data included in this article.

## Author contributions

YJ developed the research structure of the article, determined the research methodology and organized the survey and analysis. XZ designed the questionnaire, constructed the model and analyzed the data. SL summarized the research background and theory. ZW participated in the formulation of the research findings and countermeasures. YD distributed the questionnaire and collected the data. All authors contributed to the article and approved the submitted version.

## Funding

This work was supported by Jiangsu Province Postgraduate Research Innovation Program (KYCX21___0850), Nanjing University of Posts and Telecommunications Project (JG00715JX89), Nanjing University of Posts and Telecommunications Twelfth Five-Year Research Project in Education Science (GJS-XKT1415) to 2021, and Jiangsu Modern Information Service Industry Decision-making and Consulting Research Base Open Project (NYJD217011) to 2017.

## Conflict of interest

The authors declare that the research was conducted in the absence of any commercial or financial relationships that could be construed as a potential conflict of interest.

## Publisher’s note

All claims expressed in this article are solely those of the authors and do not necessarily represent those of their affiliated organizations, or those of the publisher, the editors and the reviewers. Any product that may be evaluated in this article, or claim that may be made by its manufacturer, is not guaranteed or endorsed by the publisher.
